# Pep1, a Secreted Effector Protein of *Ustilago maydis*, Is Required for Successful Invasion of Plant Cells

**DOI:** 10.1371/journal.ppat.1000290

**Published:** 2009-02-06

**Authors:** Gunther Doehlemann, Karina van der Linde, Daniela Aßmann, Daniela Schwammbach, Alexander Hof, Amitabh Mohanty, David Jackson, Regine Kahmann

**Affiliations:** 1 Max Planck Institute for Terrestrial Microbiology, Marburg, Germany; 2 Cold Spring Harbor Laboratory, Cold Spring Harbor, New York, United States of America; University of Melbourne, Australia

## Abstract

The basidiomycete *Ustilago maydis* causes smut disease in maize. Colonization of the host plant is initiated by direct penetration of cuticle and cell wall of maize epidermis cells. The invading hyphae are surrounded by the plant plasma membrane and proliferate within the plant tissue. We identified a novel secreted protein, termed Pep1, that is essential for penetration. Disruption mutants of *pep1* are not affected in saprophytic growth and develop normal infection structures. However, *Δpep1* mutants arrest during penetration of the epidermal cell and elicit a strong plant defense response. Using Affymetrix maize arrays, we identified 116 plant genes which are differentially regulated in *Δpep1* compared to wild type infections. Most of these genes are related to plant defense. By *in vivo* immunolocalization, live-cell imaging and plasmolysis approaches, we detected Pep1 in the apoplastic space as well as its accumulation at sites of cell-to-cell passages. Site-directed mutagenesis identified two of the four cysteine residues in Pep1 as essential for function, suggesting that the formation of disulfide bridges is crucial for proper protein folding. The barley covered smut fungus *Ustilago hordei* contains an ortholog of *pep1* which is needed for penetration of barley and which is able to complement the *U. maydis Δpep1* mutant. Based on these results, we conclude that Pep1 has a conserved function essential for establishing compatibility that is not restricted to the *U. maydis* / maize interaction.

## Author Summary

For many fungi that infect plants, successful invasion is coupled to a series of differentiation steps that are necessary to breach the plant cuticle. Such fungi form specialized infection structures which allow direct penetration of the plant cuticle. The smut fungus *Ustilago maydis* establishes a biotrophic interaction with its host plant maize in which the infected host cells stay alive. During biotrophic growth, the intracellularly growing hyphae are encased by the host plasma membrane. We show here that a small effector protein, which is secreted by fungal hyphae during penetration, is absolutely essential for fungal entry into plant cells. When this effector is absent, hyphal cells penetrate the plant cell wall and invagination of the plant plasma membrane is observed, but any further fungal development is arrested. This arrest coincides with the induction of massive plant defense responses. Thus, this effector, which is conserved in related fungal species, plays an essential role in suppression of plant defense responses and is critical for establishing compatibility. This is the first example where a single effector protein assumes such a crucial role for infection-related development in a plant pathogenic fungus.

## Introduction

The initial step of pathogenic development for both necrotrophic and biotrophic fungal pathogens is the successful penetration of the plant surface. Penetration can occur directly via specialized infection structures, called appressoria, which promote the localized secretion of plant cell wall degrading enzymes or build up turgor and allow penetration through mechanical force. Alternatively, fungal pathogens may use natural openings like stomata or wounds for entry [1]. The infection strategy does not appear to be linked to the subsequent lifestyle of the fungal pathogen, i.e. necrotrophs like *Botrytis cinerea* as well as hemibiotrophs such as *Colletotrichum ssp.* and *Magnaporthe grisea* directly penetrate the plant surface via appressoria [2]–[4]. Some biotrophs like most rust fungi invade plant tissue via stomata, while other biotrophs like the smut fungi and the powdery mildew fungi form appressoria that allow direct entry into the plant epidermis [5],[6].

Necrotrophic pathogens kill the invaded cell by secretion of toxic compounds or induction of reactive oxygen species (ROS), and subsequently feed on dead plant material. In biotrophic interactions and during the initial stages of hemibiotrophic interactions the infected plant cell stays alive. In such interactions, the plant plasma membrane is invaginated and encases the infecting hyphae, thereby forming a biotrophic interface. This interface, which can be established by intracellularly growing hyphae or by specialized structures (haustoria), provides nutrients to the pathogen and facilitates exchange of signals maintaining the interaction [5],[7]. Compatibility in a biotrophic interaction requires the pathogen to overcome basal plant defense responses that are elicited by recognition of conserved pathogen associated molecular patterns (PAMPs) and which can lead to pathogen arrest [8]. This initial PAMP-triggered immunity needs to be overcome by successful pathogens that use secreted effectors to interfere with these processes, and use such effectors to trigger susceptibility. Effectors may also be specifically recognized by R proteins, leading to effector triggered immunity which is often associated with cell death [8].

Haploid *U. maydis* cells mate on the leaf surface and the resulting dikaryon switches to filamentous tip growth. The growing tip cell is separated from the older parts of the hypha by a septum, and the older septated hyphal parts appear empty and are often collapsed [9]. The need of two compatible wild type strains complicates generation of deletion mutants. Therefore, the solopathogenic strain SG200 [10], which is a haploid strain engineered to carry composite mating type loci is frequently used. This strain forms filaments on the maize surface and causes disease without prior mating. On the leaf surface, SG200 as well as the dikaryon formed after mating of two compatible haploid wild type strains, develops non-melanized appressoria that directly penetrate the host tissue and establish a biotrophic interaction. Gene-for-gene systems, i.e. effectors that are specifically recognized by cognate resistance genes of the plant have not been described in this pathosystem. After penetration *U. maydis* grows intracellularly and during this stage the hyphae are surrounded by the host plasma membrane [9],[11]. *U. maydis* does not develop haustoria [12] and the intracellular hyphae pass from one cell to the next. At later stages fungal hyphae accumulate in mesophyll tissue and are found mostly in apoplastic cavities that arise in the developing tumors [13]. In these tumors plant cells enlarge, undergo mitotic divisions and the hyphal aggregates differentiate into spores.

The genome sequence of *U. maydis* revealed that this organism is poorly equipped with plant cell wall degrading enzymes [10], which is in line with its biotrophic life style where the infected plant cells stay alive. However, *U. maydis* codes for a large set of novel secreted effectors [14] and many of the respective genes are arranged in clusters [10]. During biotrophic development, the majority of these clustered effector genes is upregulated [10]. 12 of these gene clusters encoding secreted proteins were deleted and five of the respective mutants were significantly altered in virulence. Deletion of one cluster resulted in increased virulence, while mutants of the four other clusters were attenuated in virulence and showed defects at different stages of pathogenic development [10]. However, none of these clusters was reported to be essential for the initial step of biotrophic development, the penetration of epidermal cells.

Based on these studies it became clear that the repertoire of effectors with a function during disease was unlikely to be restricted to effectors whose genes reside in clusters. We have therefore initiated a systematic analysis of effector genes in *U. maydis* which is solely based on two criteria: the protein should carry a secretion signal and the predicted product should be novel i.e. should not match data base entries. Here we describe one of these novel effectors, Pep1 (Protein essential during penetration 1).

## Results

### Identification of Pep1

**Figure 1:**
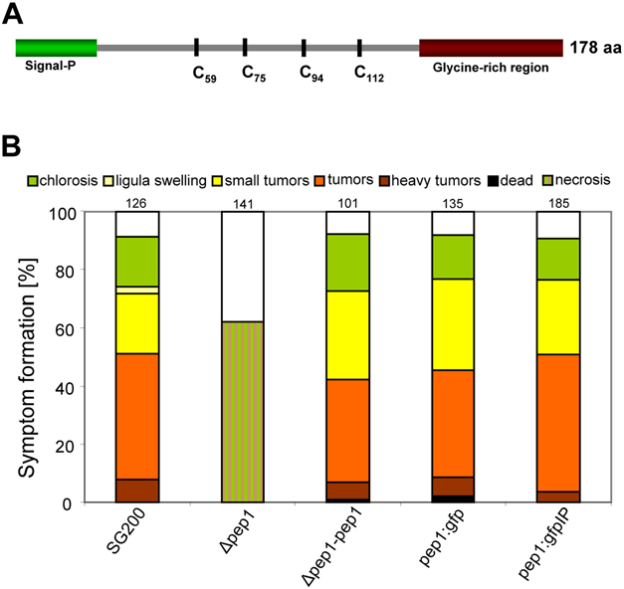
*pep1* is essential for pathogenic development of *U. maydis*. A: Predicted structure of Pep1. The protein comprises 178 aa. Signal-P (http://www.cbs.dtu.dk/services/SignalP/) predicts a putative N-terminal secretion signal (aa 1–26). In the central part of the protein four cysteine residues are present (C59, 75, 94, 112). The C-terminal part is enriched in glycine residues (aa 141–178). B: Disease rating of Early Golden Bantam maize plants 12 dpi after infection with *U. maydis* strains SG200, SG200Δpep1 (Δpep1), SG200Δpep1-pep1 (Δpep1-pep1), SG200pep1:gfp (pep1:gfp) and SG200Δpep1-pep1:gfpIP (pep1:gfpIP). Numbers indicate the total number of plants infected in three independent experiments. For details of the disease rating see Materials and Methods.

The *pep1* gene (*um01987*) resides on chromosome 3 of the *U. maydis* genome. *pep1* is not part of a gene cluster, i. e. upstream we find a putative oxidoreductase (*um01988*) and downstream a sterol carrier (*um01986*), two proteins not predicted to be secreted. The Pep1 protein comprises 178 aa and is expected to be cleaved behind a putative N-terminal secretion signal (Figure 1A). Pep1 lacks known sequence motifs associated with enzymatic function and also lacks paralogs in the *U. maydis* genome as well as homologs in known published genome sequences.

To study the function of *pep1*, gene deletions were generated in the solopathogenic strain SG200 [10]. To elucidate whether Pep1 is needed for growth of *U. maydis*, SG200 and SG200Δpep1 strains were grown under conditions of nutrient deprivation, cell wall stress or oxidative stress. In addition, filamentation was tested on charcoal containing plates (Figure S1). Under none of the tested conditions we could detect differences between these four strains, illustrating that *pep1* is not affecting growth under these conditions (Figure S1). To show that Pep1 is secreted we generated strain SG200Δpep1oma:pep1-GFP in which pep1-GFP is expressed from a strong constitutive promoter [15]. Using GFP specific antibodies, the full-length fusion protein was detected in the supernatant while supernatants of SG200 did not show a signal (Figure S4A).

Next, SG200Δpep1 was assayed for pathogenicity. The deletion of *pep1* resulted in complete loss of tumor formation (Figure 1B and Table S3). To demonstrate that the mutant phenotype resulted from disruption of *pep1*, the *pep1* gene was introduced in single copy into the *ip* locus [16],[17] of strain SG200Δpep1. The resulting strain SG200Δpep1-pep1 was fully pathogenic and showed disease ratings similar to SG200 (Figure 1B), indicating successful complementation.

### SG200Δpep1 is unable to penetrate maize epidermis cells

To examine at which stage of pathogenic development SG200Δpep1 is defective, we followed appressorium formation on inoculated maize leaves. For proper quantification of appressorium formation, GFP fluorescence of the AM1 marker, which is specifically upregulated in the hyphal tip cell forming an appressorium, was monitored [18]. 24 hpi SG200 and SG200Δpep1 strains had switched to filamentous growth and about 20% of SG200 filaments (19.73%±5.21; n = 1039) and a comparable percentage of SG200Δpep1 cells (19.76%±2.48; n = 1643) had developed appressoria. This demonstrates that the differentiation of appressoria does not require *pep1*. In addition, 48 hpi we observed that a small percentage of SG200Δpep1 cells had engaged in multiple penetration attempts (see below, Figure 3C), which was never observed after infections with SG200. Furthermore, the multiple penetration attempts seen in the *pep1* mutant suggest a defect in invasion of host tissue.

**Figure 2:**
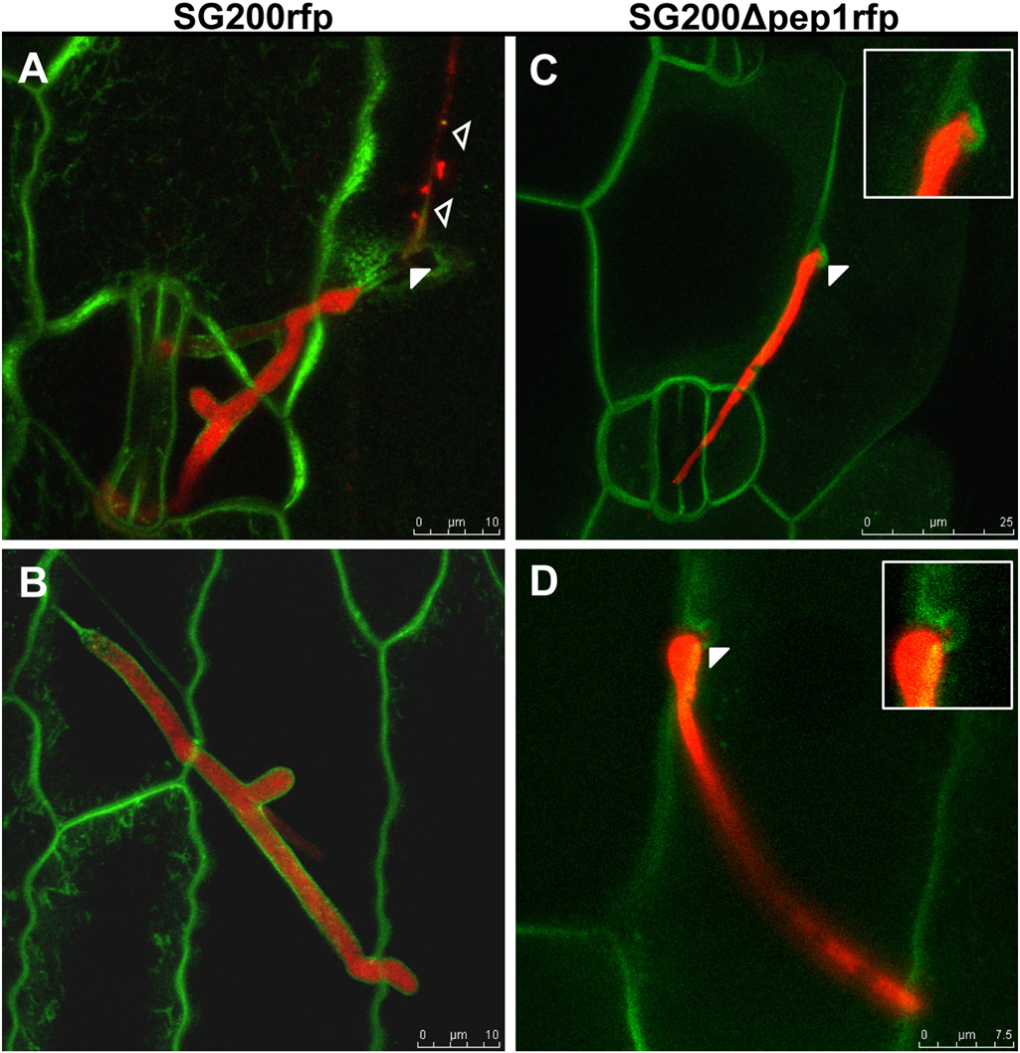
Microscopic analysis of early infection-related development of *U. maydis Δpep1* strains. Pathogenic development of SG200rfp and SG200Δpep1rfp was visualized 24 hpi on maize leaves expressing PIN1-YFP. A: SG200rfp (red) penetrated the epidermis (arrowhead) and shows hyphal branching inside epidermis cells. Open arrowheads: Empty section of penetrated hyphae on the leaf surface. B: SG200rfp grows intracellularly in the epidermal layer, being completely encased by the plant plasma membrane (green). C, D: SG200Δpep1rfp hyphae grow on the leaf surface but fail to invade epidermis cells. Mutant hyphae are arrested immediately upon penetration of the plant cell wall (arrowheads and inserts: hyphal tips of SG200Δpep1rfp invaginate the plant plasma membrane at attempted sites of penetration). Bars are given.

To analyze this presumed invasion defect in detail, we used confocal microscopy to visualize the fungus in infected leaf tissue. For a better visualization of the infection process we infected the maize line ZmPIN1a-YFP that expresses a YFP-tagged version of the PIN1a protein which locates to the plant plasma membrane [19]. Fungal hyphae growing on and inside the plant tissue were detected by cytoplasmic expression of RFP under control of the *otef* promoter in the respective strains. At 24 hpi SG200rfp hyphae were already detected in epidermal cells and were encased by the plant plasma membrane. Since the fungal cytoplasm moved into the intracellularly growing hyphae, hyphal sections on the leaf surface did not contain cytoplasm any more (Figure 2A,B). 24 hpi, hyphae of SG200Δpep1rfp could not be detected inside epidermal cells; instead, mutant hyphae were arrested immediately after penetration of the epidermal cell wall. The plant plasma membrane was found to be invaginated around mutant hyphal tips; however, no progression of mutant hyphae into the lumen of the epidermal cell was observed (Figure 2C,D). To test whether the *Δpep1* phenotype is also evident when haploid wild type strains are used instead of the solopathogenic SG200 strain, the *pep1* gene was deleted in the two compatible *U. mayd*is wild type strains FB1 and FB2 [20]. Maize plants infected with a mixture of FB1 and FB2 as well as a mixture of the deletion strains FB1Δpep1and FB2Δpep1 were analyzed by confocal microscopy 24 hpi. To visualize hyphae, they were stained by WGA-AF488, plant structures were stained with propidium iodide. Similar to SG200Δpep1, the FB1Δpep1/FB2Δpep1 dikaryon formed appressoria but penetration of epidermal cells was blocked after a short peg had entered the host cell (Figure S3). However, in rare cases, thin hyphae were found to grow into the epidermis cells and these plant cells then collapsed (Figure S3C,D). Together, this shows that the deletion of *pep1* results in a complete block of pathogenic development at the stage of host penetration.

### SG200Δpep1 induces various plant defense responses

**Figure 3:**
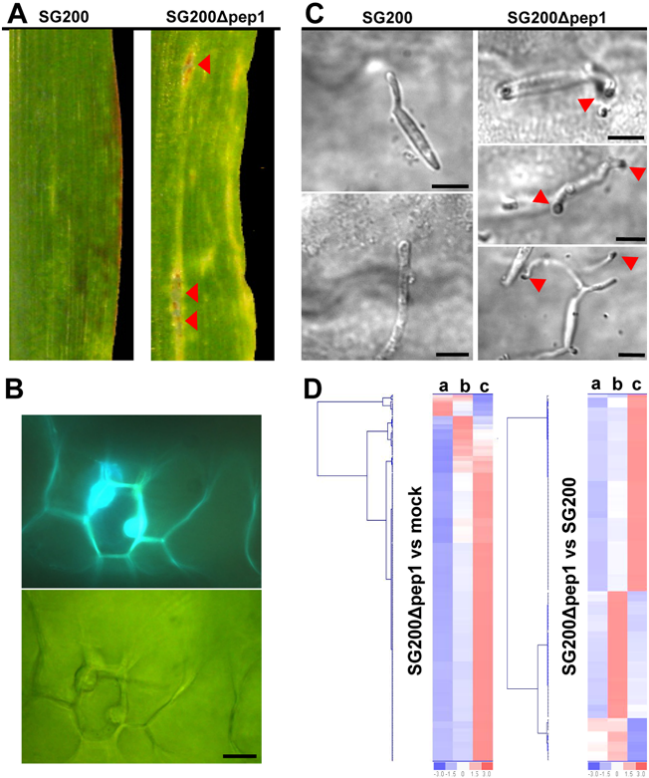
Plant responses elicited by infection with SG200Δpep1. A: Macroscopic symptoms on maize leaves 4 dpi with SG200 and SG200Δpep1. Red arrowheads mark necrotic regions in SG200Δpep1 infected leaf tissue. B: Papilla formation in maize cells attacked by SG200Δpep1. Upper panel: Cell wall autofluorescence. Lower panel: Bright field projection of the same cell. Bar: 20 µm. C: H_2_O_2_ accumulation at penetration sites was visualized by DAB staining; 48 hpi. Left panel: SG200 appressoria do not induce H_2_O_2_ accumulation. Right panel: Penetration attempts of SG200Δpep1 are accompanied by a local accumulation of H_2_O_2_ (red arrowheads). Some SG200Δpep1 hyphae display multiple penetration attempts (lower right panel). Since SG200Δpep1 cells penetrate the cell wall the DAB stain accumulates in a focal plane below the fungal cell while the hyphae are still on the leaf surface, which explains the limited sharpness of these images. Bars: 5 µm. D: Hierarchical clustering of differentially regulated maize transcripts 24 hpi with SG200Δpep1. Colors represent expression levels for each gene being above (red) or below (blue) the mean expression level (white) in mock infected tissue (a), SG200 infected tissue (b) or SG200Δpep1 infected tissue (c).

Leaf areas infected with *U. maydis* SG200 showed visible symptoms such as chlorosis, anthocyanin accumulation and small, primary tumors 4 dpi. In rare cases, small necrotic spots representing small clusters of dead cells developed (Figure 3A; [21]). In contrast, leaves infected with SG200Δpep1 did not show chlorosis but displayed large necrotic areas 4 dpi (Figure 1A, Figure 3A). Already 48 hpi SG200Δpep1 infected plants reacted with strong cell wall autofluorescence and formation of large papillae (Figure 3B). In addition, accumulation of H_2_O_2_ could be shown by staining with diamino-benzidine (DAB) at sites where SG200Δpep1 attempted to penetrate while it was absent around appressoria of SG200 (Figure 3C; [22]).

To obtain a more comprehensive picture of the plant responses induced by the *Δpep1* mutant, we performed microarray analyses of infected leaf tissue. In a previous study, the transcriptional responses of maize after infection with *U. maydis* strain SG200 have been described [21]. Using identical experimental conditions, we now compared expression profiles of SG200 infected leaves to SG200Δpep1 infected tissue 24 hpi using the Affymetrix maize genome array. At this stage, SG200 starts to establish the biotrophic interaction which goes along with a down-regulation of various defense-related genes [21]. In SG200 infected plants 24 hpi 116 genes were differentially regulated compared to mock-infections [21]. In contrast, in SG200Δpep1 infected plants 220 maize genes were found to be differentially regulated compared to mock-infected control tissue at the same time point (Table S1). In line with this, the expression of 110 maize genes was found to be significantly different (fold change ≥2) in SG200Δpep1 infected tissue compared to SG200 infected tissue (Figure 3D, Table S2). In particular, defense related genes like PR6b (Zm.791.1.S1_s_at), an endochitinase (Zm.16805.8.S1_at) and terpene synthase 6 (Zm.14496.1.A1_at) were strongly induced by SG200Δpep1 while in infections with SG200 the expression of these genes was already attenuated at this time point [21]. Interestingly, several genes associated with jasmonate biosynthesis like the lipoxygenase LOX1 (Zm.3303.1.A1_at) as well as several serine protease inhibitors that are typically activated by jasmonic acid (JA) [23] lack transcriptional induction in response to SG200Δpep1. Induction of JA signaling is a typical feature of compatible biotrophic interactions [24], i.e. its absence is therefore likely to indicate that the *pep1* mutant is incapable of establishing a biotrophic interaction.

### Pep1 is expressed during the biotrophic phase and is secreted to the apoplast

To follow expression and localization of Pep1 during different developmental stages, the coding region of *gfp* was fused to the C-terminus of Pep1. By homologous recombination, wild type *pep1* was replaced by *pep1:gfp* resulting in strain SG200pep1:gfp. In addition, a strain was generated in which *pep:gfp* was introduced into the *ip* locus of SG200Δpep1 (SG200Δpep1-pep1:gfpIP). As shown in Figure 1B, these strains were indistinguishable from SG200 with respect to causing disease, indicating that the C-terminal fusion of GFP to Pep1 did not impair its function.

**Figure 4:**
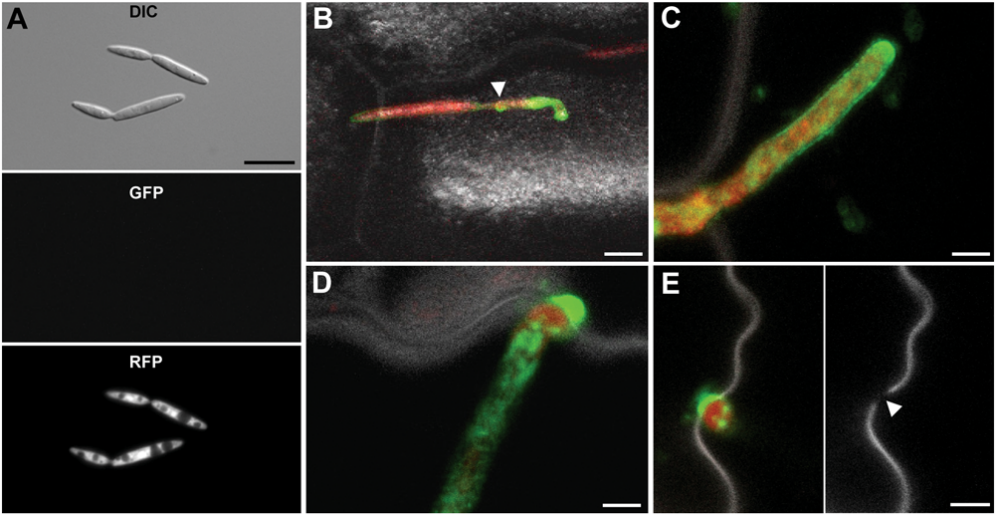
Expression and secretion of Pep1. A: Haploid sporidia of strain SG200pep1:gfpR grown in YEPSL express RFP while Pep1-GFP fluorescence is not detectable. Bar: 5 µm. B: SG200pep1:gfpR penetrating a maize epidermis cell; 24 hpi. The Pep1-GFP signal demarcates the point of penetration and becomes visible in the intracellular hyphal part (arrow). Bar: 5 µm; C: Intracellular growing hyphae of SG200pep1:gfpR showing Pep1-GFP secretion around the tip region; 48 hpi. Bar: 2 µm. D: Tip of intracellularly growing hypha of SG200pep1:gfpR during cell to cell passage. Pep1-GFP strongly accumulates at penetration sites. E: Left panel shows SG200pep1:gfpR during cell to cell passage, 48 hpi. Right panel shows the rupture of the cell wall of the same cell inflicted by the penetrating fungal hyphae (arrow); Bars: 2 µm. Pictures A, B and C are maximum projections of confocal stacks. Green: Pep1-GFP; red: RFP; grey: plant cell wall autofluorescence induced by UV-laser. In D a confocal snapshot of a single optical layer is shown.

To follow expression of *pep1:gfp* during growth, strain SG200pep1:gfp was modified to additionally express cytoplasmic RFP under control of the *otef* promoter. In SG200pep1:gfpR, no GFP fluorescence could be detected during growth in liquid culture, whereas RFP was detected in the cytoplasm of all cells (Figure 4A). When SG200pep1:gfpR was inoculated to maize leaves, Pep1-GFP expression appeared for the first time in penetrating hyphae (Figure 4B). During intracellular growth, Pep1-GFP accumulated in a slightly uneven pattern around growing hyphae (Figure 4C), at hyphal tips and particularly strong at hyphal tips during cell to cell passages (Figure 4D,E). In addition, some intracellular fluorescence was detected which is likely to reflect Pep1 during processing through the ER (Figure 4C,D). During tumor formation, i.e. 5–8 dpi, when *U. maydis* grows mainly intercellularly, Pep1-GFP could not be detected any more (not shown). In addition, expression of *pep1* was monitored by quantitative RT-PCR. In accordance to the microscopic observations, *pep1* was not detected in axenic culture while the gene was expressed at the penetration stage 18 hpi (Figure S2). During biotrophic growth, high expression levels were detected at all timepoints tested from 2–8 dpi (Figure S2).

**Figure 5:**
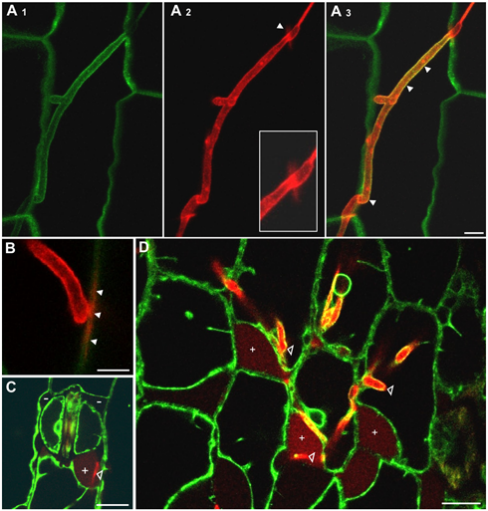
Secretion of Pep1-mCherry into the maize apoplast. A, B: SG200pep1M growing intracellularly in epidermal cells of maize line ZmPIN1a-YFP, 48 hpi. A_1_, A_2_ and A_3_ show the same hyphae with PIN1-YFP (green), Pep1-mCherry (red) and the merged yellow signals indicating co-localisation (arrowheads) around fungal hyphae, respectively. At sites of cell-to-cell passages, Pep1-mCherry is spreading from the fungal hyphae (A_2_, insert; B). Bars: 5 µm. C, D: SG200pep1M growing intracellularly in epidermal cells of maize line ZmTIP-YFP, 48 hpi. Plasmolysis was induced by 1 M NaCl, collapse of vacuoles results in enlarged apoplastic spaces. In cells colonized by SG200pep1M, this space is filled by Pep1-mCherry (+) which is not the case in cells not colonized by the fungus (−). Bars: 15 µm.

Due to autofluorescence of maize cell walls especially at penetration sites and in tumor tissue [12],[21] interference with the secreted GFP signal cannot be excluded. To overcome this problem, *pep1* under control of its own promoter was fused to the *rfp* derivate *mcherry* [25] and introduced into the *ip* locus of strain SG200Δpep1. Maize infections with the resulting strain SG200Δpep1-pep1M showed that the Pep1-mCherry fusion-protein was fully functional (not shown). SG200Δpep1-pep1M was used to infect maize lines ZmPIN1a-YFP and ZmTIP1-YFP expressing either PIN1-YFP as plasma membrane marker or TIP1-YFP, an aquaporin localizing to the tonoplast membrane (http://maize.tigr.org/cellgenomics/index.shtml). The Pep1-mCherry fusion protein was detected around intracellular hyphae, where it partially co-localized with the PIN1-YFP signal (Figure 5A). At cell to cell passages of hyphal cells, Pep1-mCherry was observed to spread between the plasma membranes of adjacent cells (Figure 5A,B). As we could not discriminate between Pep1-mCherry being localized in the plant cell wall or in the apoplastic space, plasmolysis of infected tissue was induced to enlarge the space around intracellularly growing hyphae. After plasmolysis Pep1-mCherry showed an even distribution in the now enlarged apoplastic space. mCherry fluorescence was not observed in cells which were not colonized by *U. maydis* (Figure 5C,D).

**Figure 6:**
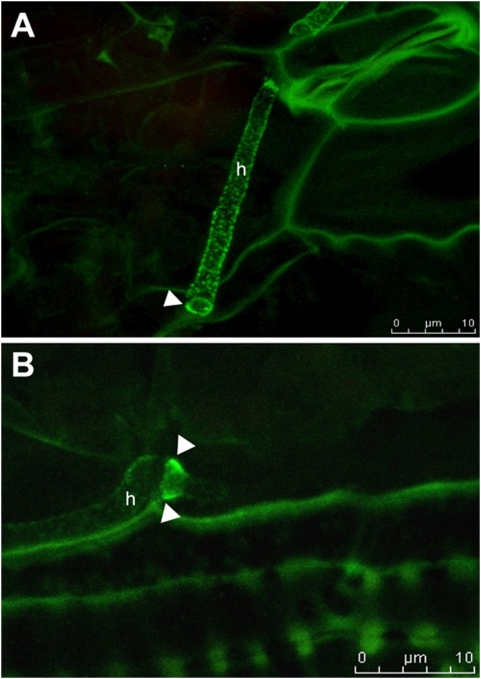
Immunolocalization of Pep1-HA in *U. maydis* infected maize tissue. A, B: Confocal projections showing immunolocalization of Pep1-HA in maize tissue infected by SG200Δpep1-pep1HA. Pep1-HA is detected around intracellular hyphae (h), predominantly accumulating at sites of cell to cell passage (arrowheads). Bars are given.

In addition to life cell imaging, strain SG200Δpep1-pep1:HA was generated and used for *in situ* immunolocalization of Pep1. Similar to what has been observed with Pep1 fused to fluorescence tags, the protein was detected on the surface of intracellularly growing hyphae (Figure 6A,B) and had a patchy distribution. The strongest accumulation of Pep1-HA was observed at sites where fungal hyphae traversed from one plant cell to the next, consistent to what has been observed with fluorescently tagged Pep1. Pep-HA could be isolated from infected maize leaves by immunoprecipitation with HA-specific antibodies and was found to be of the expected size (Figure S4B). To isolate Pep1-mCherry from infected tissue, strain SG200Δpep1-pep1:MHA was generated in which Pep1-mCherry carries an additional C-terminal HA tag. Western blot analysis of the immunoprecipitated protein revealed a signal at the expected size of the full length fusion protein. In addition, two smaller fragments were detected (Figure S4B,C).

### Pep1 is needed also for hyphal cell to cell passage

**Figure 7:**
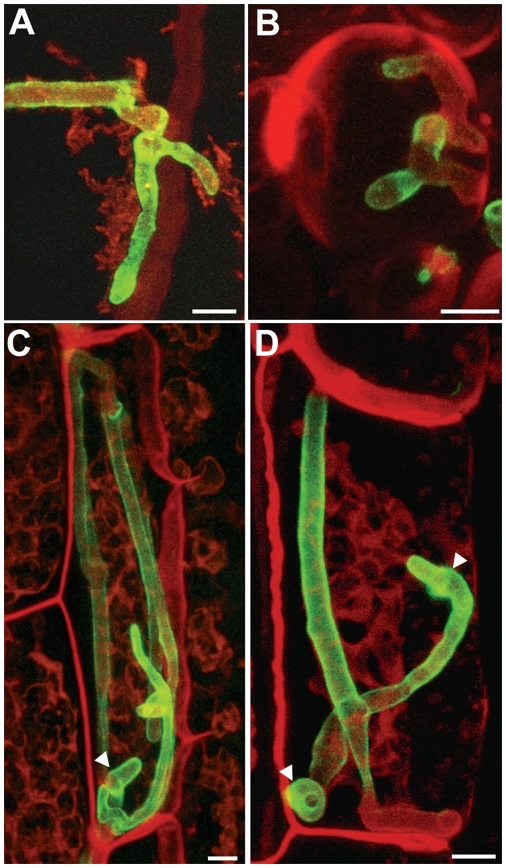
Intracellular growth of a strain expressing *pep1* under control of the *otef* promoter 7 dpi. A, B: Hyphae of SG200Δpep1otef:pep1-gfp grow intracellularly, demonstrating functionality of Pep1-GFP driven by the *otef* promoter. C, D: Insufficient expression of Pep1-GFP leads to intracellular entrapment of fungal hyphae, that show multiple, unsuccessful attempts to leave the infected cell (arrowheads). Bars: 5 µm.

Since SG200Δpep1 is blocked already upon penetration of the leaf epidermis, the mutant could not provide information concerning a role of Pep1 at later stages of the interaction between *U. maydis* and its host. To address this, we infected maize plants with *U. maydis* expressing *pep1-gfp* under control of the *otef* promoter (strain SG200Δpep1otefpep1:gfp). The artificial *otef* promoter exhibits strong, constitutive expression in haploid sporidia, penetrating filaments and during the early biotrophic phase of *U. maydis* but is shut down during the late biotrophic stage of *U. maydis* (G.D., unpublished observation). SG200Δpep1otefpep1:gfp was able to penetrate and grow intracellularly, demonstrating that expression of *pep1* under the *otef* promoter rescued the penetration defect of the *pep1* mutant (Figure 7A,B). However, tumor formation was only partially rescued; visible symptoms caused by this strain were mainly anthocyanin production, chlorosis as well as necrosis and only very small tumors were observed (Table S3). Microscopic analysis of SG200Δpep1otefpep1:gfp infected leaves 7 dpi revealed an accumulation of fungal hyphae inside plant cells. Such hyphae displayed multiple appressorium-like structures indicating unsuccessful penetration attempts (Figure 7C,D). From these results we conclude, that *pep1* is not only needed for primary penetration of the leaf epidermis, but plays an essential role for cell-to-cell passage during the intracellular phase of biotrophic growth.

### Pep1 function is conserved in the *Ustilago hordei* / barley interaction

**Figure 8:**
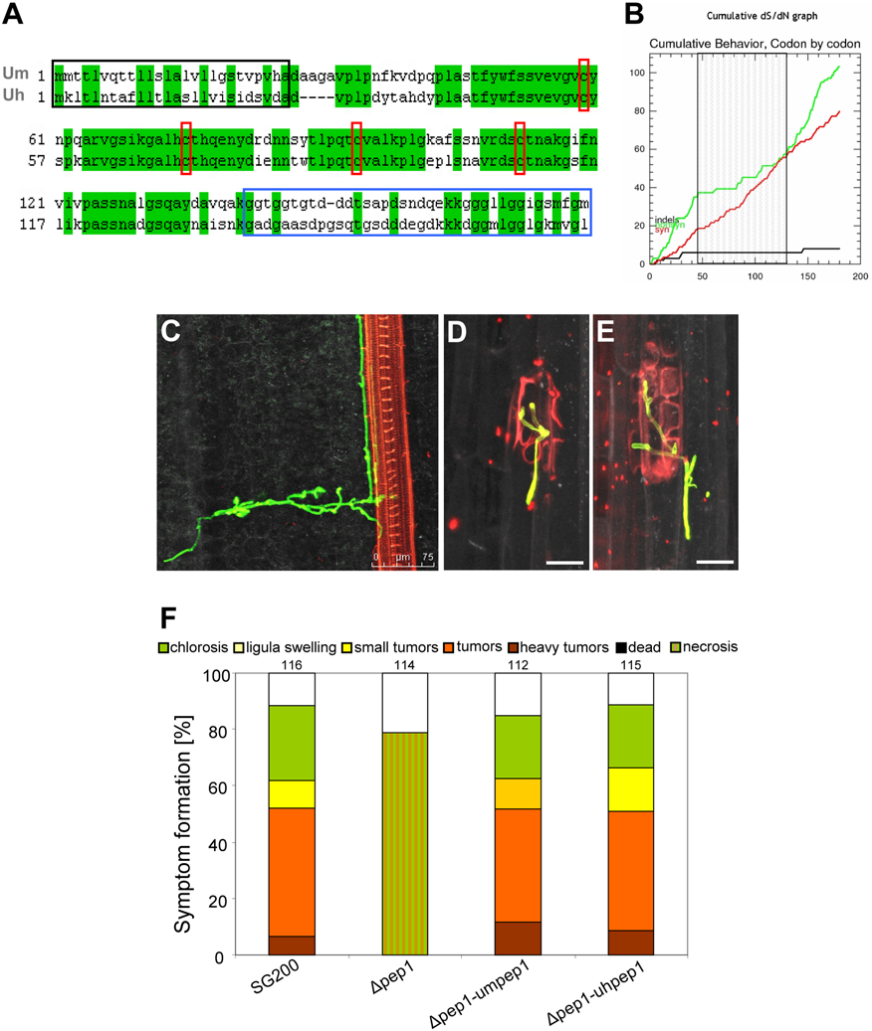
Pep1 is conserved among *U. maydis* and *U. hordei*. A: Sequence alignment of *U. maydis* Pep1 (Um) and *U. hordei* Pep1 (Uh). Identical amino acids are highlighted in green. Red boxes: conserved cysteine residues; black box: putative N-terminal secretion signal; blue box: poorly conserved glycine-rich C-terminal region. B: Cumulative plot of synonymous (red line) / non-synonymous (green line) substitutions in Pep1 from *U. maydis* and *U. hordei*. Calculation was done using the SNAP software tool (http://www.hiv.lanl.gov/content/sequence/SNAP/SNAP.html). Whereas the N-terminal and C-terminal parts of the proteins show a high ratio of non-synonymous substitutions, the central part of Pep1 (hatched box) shows a preference for sequence conservation. C–E: Confocal maximum projections of *U. hordei* 4 dpi in Golden Promise barley plants. C: Hyphae of strains 4875-5 crossed with 8a inside the leaf tissue. Hyphae (stained by WGA-AF488; green) show directed growth towards a vascular bundle (stained by propidium iodide, red). D, E: Infection by *U. hordei Δpep1* strains 4 dpi (8aΔpep1×4875-5Δpep1) reveals successful penetration into epidermal cell, collapse of the invaded epidermis cell and no further proliferation in the plant tissue. Hyphae were stained by WGA-AF488 (green); dead plant cells are stained by propidium iodide (red). Bars correspond to 25 µm. F: Disease rating of Early Golden Bantam maize plants 12 days after infection with *U. maydis* strains SG200, SG200Δpep1 (Δpep1), SG200Δpep1-pep1 (Δpep1-umpep1) and SG200Δpep1-uhpep1 (Δpep1-uhpep1). Abbreviations of the respective strain designations are given in brackets. Numbers indicate the total number of plants infected in three independent experiments. The categories for the disease rating are given above. For details of the disease rating see Materials and Methods.

After 454 sequencing of the genome of the barley covered smut fungus *Ustilago hordei* (J. Schirawski and R. Kahmann, unpublished) we identified an ortholog of *pep1* that shows 61% identity to *U. maydis* Pep1. Both proteins have an N-terminal secretion signal as well as four cysteine residues whose spacing is conserved (Figure 8A). Calculation of the ratio of synonymous to non-synonymous substitutions (ds/dn) (http://www.hiv.lanl.gov; [26]) between Pep1 of both organisms revealed a ds/dn ratio of 4.67, indicating a preference for amino acid conservation. This is particularly true for the central part of the protein that contains the conserved cysteine residues (Figure 8B). To investigate whether Pep1 is also required for penetration in *U. hordei*, *pep1* was deleted in the compatible *U. hordei* strains 4875-5 (Mat1) and 8a (Mat2). Four days post infection of barley seedlings, growth of wild type and mutant strains was analyzed by confocal microscopy. After penetration, the *U. hordei* wild type strains displayed directed growth towards the vascular bundles (Figure 8C). The *U. hordei* Δ*pep1* strains also managed to enter epidermal cells (Figure 8D,E), but proliferation inside the plant tissue was never observed. Instead, the attacked epidermis cells underwent cell death which could be visualized by propidium iodide staining of disintegrated cells (Figure 8D,E).

To test whether *U. hordei pep1* can substitute for *U. maydis pep1*, the coding region of *uh-pep1* was expressed in SG200Δpep1 under control of the *um-pep1* promoter. The resulting strain was fully pathogenic towards maize (Figure 8F), which illustrates that the two proteins are exchangeable.

### Cysteine residues are essential for Pep1 function

**Figure 9:**
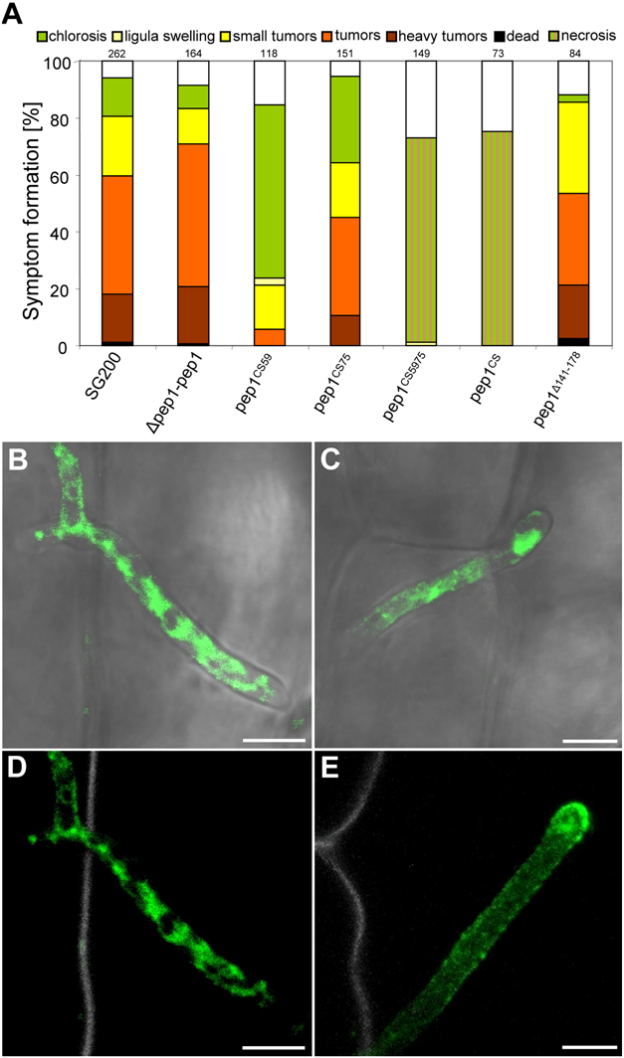
Functional analysis of Pep1. A: Disease rating of Early Golden Bantam maize plants 12 dpi with *U. maydis* strains SG200, SG200Δpep1-pep1 (Δpep1-pep1), SG200Δpep1-pep1^CS59^ (pep1CS59), SG200Δpep1-pep1^CS75^ (pep1CS75), SG200Δpep1-pep1^CS59,75^ (pep1CS59,75), SG200Δpep1-pep1^CS59,75,94,112^ (pep1CS), and SG200Δpep1-pep1^Δ141–178^ (pep1Δ141–178). Abbreviations of the respective strain designations are given in brackets. Numbers indicate the total number of plants infected in three independent experiments. The categories for the disease rating are given above. For details of the disease rating see Materials and Methods. B–E: Intracellularly growing hyphae of strain SG200-pep1:gfp^CS59,75^ (B–D) and strain SG200pep1:gfp (E), 48 hpi. Pep1^CS59,75^-GFP (D) is not secreted at the hyphal tip and accumulates inside the hyphae compared to Pep1-GFP (E). B, C: Confocal pictures showing an overlay of GFP signal (green) and bright field projection (grey). D, E: Confocal pictures showing an overlay of GFP signal (green) and UV-laser induced cell wall autofluorescence (grey). Bars correspond to 5 µm.

Pep1 does not contain conserved motifs which would allow a prediction of its mode of action. However, especially the C-terminus of *U. maydis* Pep1 is enriched in glycine residues. To test a putative function of this region, a truncated allele of *pep1* (*pep1* ^Δ141–178^) was generated. This truncated *pep1* allele was inserted in single copy in SG200Δpep1 and shown to restore wild type pathogenicity (Figure 9A). Pep1 contains four conserved cysteine residues in the central part of the protein which might be involved in formation of disulfide bridges. Mutant alleles of *pep1* were generated in which each cysteine residue of Pep1 was exchanged to serine. Mutant alleles containing substitutions in one cysteine residue (*pep1^CS59^*; *pep1^CS75^*), the first two cysteins (*pep1^CS59,CS75^*) and all four cysteine residues (*pep1^CS59,75,94,112^*) were expressed in SG200Δpep1. When single cysteine residues (C59 or C75) were substituted, pathogenicity of the respective strain was reduced (Figure 9A). The reduction was much more pronounced when C59 was mutated compared to the allele containing the C75 substitution. However, in both cases some tumors developed, indicating residual Pep1 activity (Figure 9A). Substitution of both C59 and C75 led to a complete loss of pathogenicity similar to Pep1 in which all four cysteins were replaced by serine (Pep1^CS59,75,94,112^) (Figure 9A). To disclose the reason for this essential role of the cysteine residues, a *pep1^CS59,75^:gfp* fusion was introduced in *U. maydis* strain SG200. The resulting strain SG200pep1:gfp^CS59,75^ which carries the endogenous *pep1* gene and in addition *pep1:gfp^CS59,75^* was used for maize infections. Microscopic analysis 2 dpi showed that the mutated Pep1 protein was expressed, but was found exclusively inside fungal hyphae (Figure 9B–D). This could indicate the mutant Pep1^CS59,75^-GFP being destabilized and therefore degraded immediately after secretion. However, when comparing secreted Pep1-GFP to Pep1^CS59,75^-GFP, the mutant protein was significantly enriched inside fungal cells. In addition, accumulation of the protein at the hyphal tip was absent in case of Pep1^CS59,75^-GFP (Figure 9D,E). We take this to indicate that the cysteine residues are necessary for secretion of Pep1.

## Discussion

We have shown that Pep1, a novel secreted effector protein of *Ustilago maydis*, is essential for successful invasion of maize plants.

Expression of Pep1 was not observed under axenic culture conditions and the first stage where the protein could be detected coincided with penetration. The deletion of *pep1* did not impair saprophytic development and also the overexpression of *pep1* did not cause any alterations in growth, morphology or stress resistance. However, when *pep1* was deleted *U. maydis* was unable to invade plant cells and failed to establish a compatible interaction with the host plant. In SG200Δpep1, infection-related development like filamentation and appressorium formation were unaffected. Since Δ*pep1* mutant hyphae were found to invaginate the plant plasma membrane after appressorium formation, this must indicate that lysis of the plant cell wall itself is still possible when Pep1 is absent. This was even more evident when plants were infected with a mixture of compatible FB1Δpep1 and FB2Δpep1 strains. In this case the dikaryon formed short penetration pegs and this was associated with the collapse of the invaded cell. Similarly, the dikaryon of *U. hordei* Δ*pep1* strains initially penetrated the epidermal cell but was arrested,in the penetrated cell that underwent cell death. The finding, that the *U. hordei pep1* can fully complement *U. maydis pep1* mutants shows complete functional conservation of Pep1 in the both pathosystems. The slight difference in arrest point between *U. maydis* and *U. hordei* mutants is likely to be caused by different responses or cell wall composition of the two host plants. This is also supported by the observation that *U. maydis* is arrested in the first epidermal cell when non-host barley plants are infected (G.D., unpublished). It is obvious, that both *U. maydis* and *U. hordei Δpep1*-mutants are not defective in the ability to penetrate plant cell walls but fail to establish a biotrophic interaction immediately after entry into the host plant.

Colonization of epidermal cells by biotrophic fungi requires the establishment of a biotrophic interface which mediates nutrient uptake and provides the contact zone where suppression of defense responses by the fungus takes place [7]. In infections with *U. maydis* strain SG200 early plant defense responses are induced and these are downregulated upon penetration [21]. In the absence of *pep1* this downregulation was not observed, i.e. of the 37 defense related genes which were significantly repressed in the interaction with SG200 24 hpi [21], 23 genes were found to be highly induced 24 hpi in SG200Δpep1 infected maize tissue. Another major difference concerned genes associated with JA signaling. These were strongly upregulated after infection with SG200 but not in response to SG200Δpep1 [21]. Similarly, two Bowman-Birk type trypsin inhibitors were highly induced after infection with SG200 but induction was absent after infection with SG200Δpep1. For Bowman-Birk type trypsin inhibitor genes in rice it has been demonstrated that they are transcriptionally induced by JA but repressed by salicylic acid (SA) [27]. This suggests that the typical transcriptional response to biotrophic pathogens that coincides with elevated JA levels and a repression of SA signaling [24] is not established after infections with SG200Δpep1. Moreover, production of ROS, papilla formation and the transcriptional induction of PR genes observed in response to SG200Δpep1 are typical for non-host responses in incompatible plant-pathogen interactions [28].

To understand the function of secreted effector proteins it is necessary to establish where they localize. Most extensive work on localization and function has been done on bacterial effectors which are translocated into the host cell via the type III secretion systems [29]. Remarkable advances have also been made in the oomycete field where many effectors carry a RXLR-EER motif that mediates translocation of effectors into the plant cell while a second group of effectors that lack this motif function in the apoplast [30]–[33]. The described secreted fungal effectors follow similar principles, i.e. they either have an apoplastic function or act inside the plant cell. However, the group of fungal effectors which are translocated to plant cells lack common motifs. Among these are *M. grisea* AVR-Pita, *Uromyces fabae* RTP1 and the flax rust effectors AvrM, AvrL657, AvrP123; AvrP4 [34]–[37]. From these proteins only RTP1 was directly detected inside host cells by immuno-localization [36].Transfer of the other fungal effectors was inferred from their ability to trigger cell death when expressed in the cognate resistant line or their interaction with a cytoplasmic resistance gene in yeast two-hybrid assays [7]. Apoplastic fungal effectors like *Cladosporium fulvum* effectors Avr2, Avr4 and Ecp6 have been directly isolated from apoplastic fluid of infected tomato plants and several oomycete effectors were detected in isolated apoplastic fluid after antibodies had been raised [38]–[41].

Pep1 secretion from intracellularly growing hyphae could be shown by generating biologically active GFP and mCherry fusions and this did not require overexpression. Secretion of Pep1 and accumulations at sites where hyphae passage from cell to cell was confirmed by immunolocalisation of HA-tagged Pep1 protein. However, it was impossible to determine in which plant compartment Pep1 resides because of the tight encasement of the intracellular hyphae by plant plasma membrane. This problem could be solved by inducing plasmolysis, which allowed to detect Pep1:mCherry now in the drastically enlarged apoplastic space around intracellular hyphae. By immunoprecipitation full length HA-tagged Pep1 could be isolated from infected plant tissue. This contrasts the situation in tomato where it was not possible to recover affinity-tagged secreted effectors from infected plant after overexpressing the genes via a PVX system [42]. In addition, immunoprecipitations of a mCherry-HA tagged Pep1 allowed to demonstrate that a significant amount of full-lengh fusion protein could be isolated from infected plant tissue. However, some material being significantly smaller than mCherryHA (which is therefore unlikely to show fluorescence) was detected. Another fragment of about 35 kD is indicative of processing/degradation within the Pep1 part of the fusion protein. This was not observed when immunoprecipitating Pep1-HA and therefore we consider it likely that this form was generated during protein extraction. Since this clearly shows that no cleavage of full length mCherryHA from Pep1 occurs inside the plant tissue, we conclude that the observed fragments, even if they were present in the infected tissue, should not affect the Pep1-localization shown by fluorescence microscopy. Collectively, the presented data suggest an apoplastic localization of Pep1.

The elicitation of plant defense responses typically results in the massive accumulation of PR proteins in the apoplast [43]. Many of these PR proteins have enzymatic functions and ß-1,3 glucanases or proteases can directly harm the pathogen or degrade secreted effectors with the result of disabling the pathogen. For several fungal and oomycete effectors it has been demonstrated that they target such PR proteins: The *C. fulvum* effector protein Avr2 has been shown to inhibit the apoplastic tomato proteases RCR3 and PIP1 [40],[44]; and *Phytophtora infestans* secretes several inhibitors for apoplastic proteases of tomato [38],[45],[46]. A different function has been shown for Avr4, which prevents hydrolysis of fungal cell walls by plant chitinases [47]. While the role of individual protease inhibitors for disease progression has not been analyzed in *Phytophtora*, silencing of *avr*2 and *avr*4 leads to decreased virulence of *C. fulvum* on tomato [48],[49]. Similarly the *C. fulvum* effector Ecp6 (whose function is unknown) is required for full virulence [39].

In contrast to these effectors which are virulence factors, Pep1 is essential for compatibility. When absent, *U. maydis* and *U. hordei* fail to establish a biotrophic interface. *pep1* mutants are recognized by their respective host plants and elicit defense responses that are so strong that a host now acts as if it was a non-host. This, however, does not suffice as an explanation for host specificity. In this case we would have expected that all smuts that express *pep1* should cause disease on the same host plants (which is not the case). Therefore, we propose that *pep1* affects compatibility on an early level that precedes the action of host specificity factors.

Which is the molecular function of Pep1? At present, we can only speculate about its mode of action. Pep1 of *U. maydis*, which is predicted to comprise 152 aa after signal peptide cleavage, is unrelated to proteins or functional domains of described database entries. This makes it unlikely that Pep1 has an enzymatic function. A glycine-rich domain of 37 aa at the C-terminus was deleted without affecting biological activity. This domain is considerably less conserved between *U. maydis* Pep1 and *U. hordei* Pep1 than the central domain. Given the apoplastic localization and the importance of the four cysteine residues for secretion of Pep1 we consider a compact structure of Pep1 that requires disulfide bridge formation most likely. Fungal and Oomycete plant pathogens have been shown to secrete a broad range of putative enzyme inhibitors to counteract plant hydrolases and many of these are cysteine-rich and attain their compact structure through disulfide bridge formation [33]. Among these are small cysteine-rich apoplastic proteins like Avr2, the EPI and EPIC proteins of *Phytophtora* that all target specific pathogenesis related plant proteases [38],[44],[46],[49]. Another small effector of *P. sojae* specifically targets ß-1,3-glucanases of soybean [50]. Due to selective pressure, both, the genes encoding the plant enzymes and the genes encoding the fungal/oomycete inhibitors exist in large gene families. These features were proposed to provide robustness to the systems but at the same time limit the effects of individual genes due to redundancy [33]. With respect to Pep1 these criteria do not apply, i.e. paralogous genes for *pep1* are neither found in *U. maydis* nor in *U. hordei*. We have not analyzed allelic variation, however, the degree of sequence conservation and the preference of synonymous nucleotide substitutions over non-synonymous substitutions in the central domain is remarkably high. This likely indicates that this domain adopts a defined structure that cannot be altered by mutation without affecting the function of the protein. And finally, the phenotype of *pep1* deletion is dramatic, reinforcing the absence of redundant functions. Thus, if Pep1 is an enzyme inhibitor, we would predict that it should have little or no specificity, i.e. interacts with many enzyme isoforms. Fungal effectors like the *C. fulvum* protease inhibitor Avr2 which specifically interacts with two plant proteases shows strong diversifying selection, and this is likely the consequence of preventing recognition [44]. This contrasts the situation in Pep1 where we find a high conservation of the central domain which is essential for Pep1 function. Alternatively, Pep1 could act as a kind of chaperone protecting/activating other secreted effectors or facilitate the establishment of the fungal/host interface by binding toxic compounds or interfere with plant signaling. Solving the molecular structure of Pep1 and identification of interacting molecules will help to disclose its function and the processes it interferes with. As two-hybrid screens were unsuccessful, presumably due to incorrect folding of the protein (G.D., unpublished), biochemical approaches are now under way. The understanding of how Pep1 affects plant defense responses is likely to provide fundamental new insights into the initial steps that are required for the establishment of a compatible, biotrophic interaction between fungi and their host plants.

## Materials and Methods

### Fungal strains and growth conditions

*U. maydis* SG200 [10] and its derivatives (Table 1) were grown at 28°C in YEPSL (0.4% yeast extract, 0.4% peptone, 2% sucrose) and used in plant infections as described [22]. Disease symptoms were scored 12 dpi as described previously [10]. Symptoms caused by SG200Δpep1 mutants were classified into the additional category “chlorosis/necrosis”. For growth assays, *U. maydis* strains were grown for 48 hours on plates containing CM agar supplemented with 1% glucose and various stress-inducing compounds whose concentrations are indicated (Figure S1). To induce filamentous growth, strains were cultured on PD agar containing 1% activated charcoal. *U. hordei* strains 4875-5 and 8a as well as their derivatives (Table 1) were grown under the same experimental conditions as *U. maydis*. For infection of barley plants (Golden Promise), cultures of the compatible strains were grown until an OD_600_ of 1.0 in YEPSL, and mixed prior to needle infection of barley plants 10 days post sawing.

**Table 1:** Strains used in this study.

Strain	Genotype	Reference
*Ustilago maydis:*		
SG200	a1mfa2 bW2bE1	[10]
FB1	a1 b1	[20]
FB2	a2 b2	[20]
SG200rfp	a1mfa2 bW2bE1 ipr[P*otef-rfp*]ips	[53]
SG200pep1:gfp	a1mfa2 bW2bE1 *um-pep1-egfp:hph*	This study
SG200Δpep1	a1mfa2 bW2bE1 Δ*um-pep1::hph*	This study
FB1Δpep1	a1 b1 Δ*um-pep1::hph*	This study
FB2Δpep1	a2 b2 Δ*um-pep1::hph*	This study
SG200Δpep1rfp	a1mfa2 bW2bE1 Δ*um-pep1::hph* ipr[P*otef-rfp*]ips	This study
SG200Δpep1otef:pep1	a1mfa2 bW2bE1 Δ*um-pep1::hph* ipr[P*otef-um-pep1*]ips	This study
SG200Δpep1otef:pep1-gfp	a1mfa2 bW2bE1 Δ*um-pep1::hph* ipr[P*otef-um-pep1*-*egfp*]ips	This study
SG200Δpep1oma:pep1-gfp	a1mfa2 bW2bE1 Δ*um-pep1::hph* ipr[P*oma-um-pep1*-*egfp*]ips	This study
SG200Δpep1-pep1	a1mfa2 bW2bE1 Δ*um-pep1::hph* ipr[P*wt-um-pep1*]ips	This study
SG200Δpep1-pep1:gfpIP	a1mfa2 bW2bE1 Δ*um-pep1::hph* ipr[P*wt-um-pep1-egfp*]ips	This study
SG200pep1:gfpR	a1mfa2 bW2bE1 *um-pep1-egfp:hph* ipr[P*otef-rfp*]ips	This study
SG200Δpep1-pep1M	a1mfa2 bW2bE1 Δ*um-pep1::hph* ipr[P*wt-um-pep1-mcherry*]ips	This study
SG200Δpep1-pep1HA	a1mfa2 bW2bE1 Δ*um-pep1::hph* ipr[P*wt-um-pep1*-HA]ips	This study
SG200Δpep1-pep1MHA	a1mfa2 bW2bE1 Δ*um-pep1::hph* ipr[P*wt-um-pep1-mcherry*-HA]ips	This study
SG200Δpep1-uhpep1	a1mfa2 bW2bE1 Δ*um-pep1::hph* ipr[P*wt-uh-pep1*]ips	This study
SG200Δpep1-pep1^CS59^	a1mfa2 bW2bE1 Δ*um-pep1::hph* ipr[P*wt-um-pep1* ^CS59^]ips	This study
SG200Δpep1-pep1^CS75^	a1mfa2 bW2bE1 Δ*um-pep1::hph* ipr[P*wt-um-pep1* ^CS75^]ips	This study
SG200Δpep1-pep1^CS59,75^	a1mfa2 bW2bE1 Δ*um-pep1::hph* ipr[P*wt-um-pep1* ^CS59,75^]ips	This study
SG200Δpep1-pep1^CS59,75,94,112^	a1mfa2 bW2bE1 Δ*um-pep1::hph* ipr[P*wt-um-pep1* ^CS59,75,94,112^]ips	This study
SG200-pep1:gfp^CS59,75^	a1mfa2 bW2bE1 ipr[P*wt-um-pep1* ^CS59,75:egfp^]ips	This study
SG200Δpep1-pep1^Δ141–178^	a1mfa2 bW2bE1 Δ*um-pep1::hph* ipr[*um-pep1* ^Δ141–178^]ips	This study
*Ustilago hordei:*		
4875-5	a12b1	[58]
8A	a2b2	ATCC 90511
4875-5Δpep1	a1b1 Δ*uh-pep1::hph*	This study
8AΔpep1	a2b2 Δ*uh-pep1::hph*	This study

P: promoter; *a1* and *a2*: mating type loci of *U. maydis* or *U. hordei*, *mfa2*, *bW2*, *bE1*: mating type genes; ips: *ip* allele encoding sensitivity to carboxin; ipr: *ip* allele encoding resistance to carboxin; *hph*: hygromycin B phosphotransferase.

### Plant lines

Barley plants of the variety Golden Promise were obtained from the IFZ (Giessen, Germany). Maize lines of the variety Early Golden Bantam were obtained form Olds Seeds (Madison). Maize lines ZmPIN1a-YFP and ZmTIP1-YFP were provided from Cold Spring Harbor Laboratory.

### Strain constructions

All *U. maydis* strains generated in this study are derived from the solopathogenic strain SG200 and the wild type isolates FB1 and FB2 (Table 1; [10],[20]). For the deletion of *pep1* (Gene bank accession: XP_758134) a PCR-based approach using hygromycin as resistance marker [51] was used. 1 kb of each flanking region of *pep1* were amplified by PCR using primers 5′-TTGGTGGACAGTCACGAGCATTC-3′ and 5′-TTCGGCCATCTAGGCCAC TCTGCTCGCCAGCATATCAC-3′ for the left border and primers 5′-CACGGCCTGAGTGGCCCAACTGCTTTCTGCCCTTTG-3′ and 5′-TTTCA GGGCAGCTCAGAGTG-3′ for the right border. PCR products were digested with *Sfi*I and ligated to the *hph* cassette of pBS-*hhn* [51]. For integrations into the *ip* locus of *U. maydis*, plasmids derived from p123 were used [52]. For cytoplasmic *rfp* expression under control of the *otef* promoter, p123-rfp [53] was introduced into the *ip* locus of strains SG200, SG200Δpep1 and SG200pep1:gfp, respectively. To substitute *pep1* by *pep1:gfp*, 1 kb of *U. maydis* genomic sequence containing the coding region of *pep1* was amplified by PCR as left border using primers 5′-GCAAGCCTAGCAATCTTCGATAGC-3′ and 5′-CACGGCCGCGTTGGCCCCGGTGGCGATCGAGCGCATGCCAAACATGCTACCGATTCC-3′, digested with *Sfi*I and ligated to the *gfp:hph* cassette of plasmid pUMa317 [54]. As right border, 1 kb including the terminator region of *pep1* was amplified by primers 5′-CACGGCCTGAGTGGCCGCTGCGACGTCGTTGATGATGAC-3′ and 5′-CTCCACTCAAGACTCACAGACT-3′, digested with *Sfi*I and ligated to the *gfp:hph* cassette of plasmid pUMa317. For complementation of SG200Δpep1, the *pep1* gene with its complete promoter region was amplified using primers 5′-GCAAGCTTACGACGGATGCGCTATCGTCAC-3′ and 5′-TAGCGGCCGCCTGG CGAGCAGAGTCATCATCAAC-3′ and ligated into the *Hind*III and *Not*I sites of vector p123 resulting in p123-pep1. To complement SG200Δpep1 with pep1 pep1^Δ141–178^, the truncated *pep1* coding region with its complete promoter region was amplified using primers 5′-GCAAGCTTACGACGGATGCGCTATCGTCAC-3′ and 5′-TTGCGGCCGCTTGGCTTGAACCGCATCGTAAGC-3′ and ligated into the *Hind*III and *Not*I sites of vector p123 which resulted in plasmid p123- pep1^Δ141–178^. To introduce *pep1:gfp* into the *ip* locus, plasmid p123-pep1:gfp was constructed by amplifying the *pep1* gene using primers 5′-GCAAGCTTACGACGGATGCGCTA TCGTCAC-3′ and 5′-CACCCATGGCGGTGGCGATCGAGCGCATGCCAAACA TGCTACCGATTCC-3′, and ligating the PCR product via *Hind*III and *Nco*I into p123. To express *pep1:gfp* under control of the *otef* promoter, the coding region of *pep1* was amplified using primers 5′-ATGGATCCGATGATG ACCACACTGGTGCAAAC-3′ and 5′-CACCCATGGCGGTGGCGATCGAGC GCATGCCAAACATGCTACCGATTCC-3′. The PCR product was digested with *BamH*I and *Nco*I and ligated to the respective sites in p123 resulting in plasmid p123-otefpep1:gfp. The C-terminal HA-tag was introduced by amplification of the *pep1* with primer 5′-GCAAGCTTACGACGGATGCGCTATCGTCAC-3′ and primer 5′-TAGCGGCCGCTCAGGCATAGTCGGGGACGTCGTAGGGATAGCCGCCCGACATGCCAAACATGCTACCGATTC-3′ which contains the HA-tag encoding sequence. This PCR product was digested with *Hind*III and *Not*I and ligated into p123 resulting in plasmid p123-pep1HA. To fuse *pep1* with *mcherry*, plasmid p123-mcherry was constructed by excision of the *gfp* coding region from p123 using *Nco*I and *Not*I and substitution by *mcherry* derived from plasmid pCRII-mcherry (kindly provided by G. Steinberg). Similarly, for *mcherry::HA* constructs, *mcherry* was amplified by primer 5′-CTCCATGGTGAGCAAGGGC-3′ and primer 5′-CTGCGGCCGCTTAAGCGTAATCTGGAACATCGTATGGGTACTTGTAC AGCTCGTCCATGCCGC-3′ that contains the HA sequence and introduced into the *Nco*I and *Not*I sites of p123 and subsequently fused to pep1 as described for p123-pep1:gfp. To express *U. hordei pep1* in SG200Δpep1, the coding region of *uhpep1* was amplified with primers 5′-TTGATATCAACGATGAAGCTCAC ACTCAACACCG-3′ and 5′-TTGCGGCCGCTCAGAGCCCAACCATCTTACC-3′ genomic DNA of *U. hordei* strain 4875-5. The PCR product was digested with *EcoR*V and *Not*I and ligated with *EcoR*V / *Not*I digested PCR product of primers 5′-ACCGCTGCGACGTCGTTGATGATG-3′ and 5′-GTCGAGAGTCCTCAG GATGGTTC-3′ that facilitate an inverse amplification of p123-pep1 without the *U. maydis pep1* coding region.

### Nucleic acid manipulations, quantitative real time PCR and DNA microarrays

Standard molecular techniques were used [55]. Transformation of *U. maydis* and isolation of genomic DNA was performed as described previously [56]. All generated constructs were sequenced prior to *U. maydis* transformation. Isolated *U. maydis* transformants were tested for single integration events in the desired loci by southern analysis. To substitute cysteine residues in *pep1* by serine, single point mutations were introduced in plasmid p123-pep1 using the “Quick Change Multi” site directed mutagenesis kit (Stratagene, La Jolla, USA). Introduced mutations were confirmed by sequence analysis.

For the Affymetrix microarray experiments, maize plants (Early Golden Bantam) grown in a phytochamber were infected with SG200Δpep1 as described previously and samples of infected tissue were colleted 24 hpi, 1 h before the end of the light period and directly frozen in liquid nitrogen [21]. Samples were collected in three independently conducted experiments by sampling 30 plants per experiment. For RNA isolation, material from the 30 plants was pooled, ground in liquid nitrogen and RNA was extracted with Trizol (Invitrogen, Karlsruhe, Germany) and purified using an RNeasy kit (Qiagen, Hilden, Germany).

Affymetrix Gene chip^R^ maize genome arrays were done in three biological replicates, using standard Affymetrix protocols (Midi_Euk2V3 protocol on GeneChip Fluidics Station 400; scanning on Affymetrix GSC3000). Expression data were submitted to GeneExpressionOmnibus (http://www.ncbi.nlm.nih.gov/geo/) (Accession Number: GSE12892). Data analysis was performed using Affymetrix GCOS1 1.4, bioconductor (http://www.bioconductor.org/) and dChip1.3 (http://biosun1.harvard.edu/complab/dchip/), as described (Doehlemann *et al.*, 2008b). We considered changes >2-fold with a difference between expression values >100 and a corrected p-value<0.001 as significant.

Expression of *pep1* was analyzed by qRT-PCR. RNA samples were isolated with Trizol as described above. To isolate *U. maydis* cells during the penetration stage 18 hpi from the maize leave surface, infected leaves were coated by liquid latex. The latex was dried and then peeled from the leaves. Peeled latex, containing the fungal structures extracted from the leaf surface was then used for RNA-isolation as described above. For cDNA synthesis, the SuperScript III first-strand synthesis SuperMix assay (Invitrogen, Karlsruhe, Germany) was employed, using 1 µg of total RNA. qRT-PCR was performed on a Bio-Rad iCycler using the Platinum SYBR Green qPCR SuperMix-UDG (Invitrogen, Karlsruhe, Germany). Cycling conditions were 2 min 95°C, followed by 45 cycles of 30 sec 95°C / 30 sec 61°C / 30 sec 72°C. Control gene primers for amplification of the *U. maydis* peptidylprolyl isomerise (*ppi*) were rt-ppi-for: 5′-ACATCGTCAAGGCTATCG-3′ and rt-ppi-rev: 5′- AAAGAACACCGGACTTGG-3′. To amplify a *pep1* PCR-fragment, primers rt-pep1-for: 5′- CACTGACGACGACACCT-3′ and rt-pep1-rev: 5′- TGCTACCGATTCCTCCT-3′ were used.

### Microscopy

Fungal hyphae were stained with WGA-AF 488 (Molecular Probes, Karlsruhe, Germany). Plant membranes were visualized using Propidium Iodide (Sigma): Samples were incubated in staining solution (1 µg/ml Propidium Idodide, 10 µg/ml WGA-AF 488; 0.02% Tween20) for 30 min and washed in 1× PBS (pH 7.4). Visualization of H_2_O_2_ by DAB was performed as described [22]. Confocal images were recorded on a TCS-SP5 confocal microscope (Leica, Bensheim, Germany); using WGA-AF 488: excitation at 488 nm and detection at 500–540 nm. Autofluorescence of cell wall material was excited at 405 nm and detected at 415–460 nm. For mCherry fluorescence of hyphae in maize tissue, an excitation of 561 nm and detection at 580–630 nm was used. GFP fluorescence was excited with a 488 nm laser, emission was detected at 495–530 nm. YFP fluorescence of tagged plant proteins was excited at 495 nm and detected at 510–550 nm.

### Immunoprecipitation of Pep1 from maize leaves

For immunoprecipitation of Pep1-HA and Pep1-mCherry-HA from infected maize tissue, infected areas of 60 plants were excised 3 dpi after infection with the respective *U. maydis* strains and directly frozen in liquid nitrogen. Frozen leaves were ground in liquid nitrogen, mixed with extraction buffer and centrifuged for 30 min at 28100g. All samples were adjusted to a protein concentration of 2.4 mg/ml in a volume of 7,5 ml and mixed with 10 µl HA-matrix (Roche) for 16 h at 4°C on a shaker. Elution was performed according to the HA-Kit protocol (Pierce).

### Immunodetection of Pep1

Overnight cultures of *U. maydis strains* SG200 and SG200Δpep1oma:pep1-gfp were harvest by centrifugation, washed once and were resuspended in 50 ml NM media containing 0,5% glucose to an OD_600 nm_ of 0,20 and grown at 28°C to an OD_600 nm_ of 0.80. Cells were harvest by centrifugation, the supernatant was collected and percipitatetd by TCA. Then the pellets were washed seven times with 80% icecold acetone and resuspendet in 30 µl SDS loading buffer. All protein samples were separated by SDS-PAGE and transferred to a nitrocellulose membrane. After electroblotting, filters were saturated with 5% non-fat dry milk in TBS (20 mM Tris-HCl, 137 mM NaCl, pH 7.6), 0.1% Tween for 1 hr at room temperature (RT). For detection of Pep1-GFP, a monoclonal GFP specific antibodies (Clontech, Mountain View, USA) was used (1∶10000). To detect HA-tagged proteins, a monoclonal mouse-anti-HA antibody (Sigma-Aldrich) (dilution 1∶7500) was used. As secondary antibody an anti-mouse peroxidase conjugate (1∶10000) (Sigma-Aldrich) was used. For chemiluminscence detection, ECL Plus Western Blot detection reagent (GE Healthcare) was used. For *in situ* detection of Pep1-HA, maize leaves were harvested three days after infection with SG200pep1HA. Infected tissue was treated as described previously [57]. For detection of the HA-tag, a monoclonal mouse-anti-HA antibody (Sigma-Aldrich, dilution 1∶7500) was used. As secondary antibody, anti-mouse conjugated with AF488 (Molecular Probes) was used in a 1∶5000 dilution. Confocal microscopy of the samples was done as described above. Control samples were maize leaves infected with SG200 and these were treated identical to SG200pep1HA infected tissue to verify Pep1-HA detection. In another control, SG200 infected leaves were used for detection of maize tubulin (mouse-anti-tubulin; Sigma-Aldrich, dilution 1∶7500). In both control samples, plant structures showed the same background, but no fluorescence of fungal hyphae was detected (Figure S5).

## Supporting Information

Figure S1 Growth of *U. maydis* SG200, SG200Δpep1 and SG200Δpep1otef:pep1 on growth media providing different stresses. Precultures of *U. maydis* were grown in YEPSL to an OD600 of 1.0. Cells were washed in water and recalibrated to an OD600 of 1.0 and diluted 10-fold each in four steps. From these suspensions droplets of 6 µl each were dropped on the different media. After 48 hours incubation at 28°C pictures were taken. A: PD agar containing 1% Charcoal; B: CM agar supplied with Calcofluor (100 µg/ml); C: CM agar supplied with 2 mM H_2_O_2_; D: CM agar supplied with Congored (50 µg/ml); E: Ammonium Minimal Medium; F: Nitrogen Minimal medium. I) SG200 II) SG200Δpep1 III) SG200Δpep1-pep1 IV) SG200Δpep1-otef:pep1.
(7.83 MB TIF)

Figure S2 Expression of *pep1* during pathogenic development of *U. maydis*. Quantitative real-time PCR on *pep1* expression of *U. maydis* strain SG200. Sporidia grown in axenic culture did not show detectible expression of pep1. In SG200 cells that were extracted from the maize leaf surface (18 hpi) *pep1* transcript was detected. High levels of *pep1* expression were detected in maize leaf tissue taken at different time points after infection with SG200. The strongest expression of *pep1* was observed during the early biotrophic phase (2 dpi) and during late stages of infection (6 and 8 dpi).
(5.94 MB TIF)

Figure S3 Microscopic analysis of *U. maydis* FB1/2Δpep1 mutants after inoculation on maize plants. Confocal projections showing fungal hyphae stained with WGA-AF488 (green) and plant cells stained with propidium-iodide (red) 24 hpi. A: FB1×FB2 crossings have penetrated the leaf surface (white arrowhead) and grow intracellularly. Hyphae on the leaf surface are collapsed (open arrowheads) after plant penetration. B–D: At the same time-point, the FB1Δpep1×FB2Δpep1 dikaryon was arrested immediately upon penetration similar to SG200Δpep1 (Figure 2). In addition, short hyphae of FB1Δpep1×FB2Δpep1 (left panel, C1 and D1 and insert) can be found in collapsed epidermis cells (overlay: right panel, C21 and D2). Bars are given.
(7.99 MB TIF)

Figure S4 Western detection of Pep1-GFP, Pep1-HA and Pep1-mCherry-HA. Western blot of Pep1-GFP secreted from *U. maydis* strain SG200Δpep1oma:pep1-gfp. SG200: In culture-supernatant of SG200 cells, no Pep1-GFP was detected by an anti-GFP serum. Pep1-GFP: In culture-supernatant of SG200Δpep1oma:pep1-gfp, Pep1-GFP was detected in full-length. B: Immunoprecipitation of Pep1-HA and Pep1-mCherry-HA (Pep1-MHA): HA tagged Pep1 and Pep1-mCherry were immunoprecipitated from maize tissue infected with *U. maydis* strain SG200Δpep1-pep1HA and SG200Δpep1-pep1MHA, respectively (3 dpi) using monoclonal HA-specific antibodies. SG200: From SG200 infected maize tissue, no precipitated protein was detected. Red arrows: Full length fusion protein at the expected size for Pep1-HA and Pep1-mCherry-HA. C: Schematical description of the Pep1-mCherry-HA fusion protein. Numbers: Expected molecular weight [kDa] of the individual parts of the fusion protein. SP: signal peptide (cleaved off during secretion).
(3.43 MB TIF)

Figure S5 Control samples showing specificity of anti-HA serum used for immunolocalization of Pep1-HA. A: Confocal projection showing unspecific fluorescence of *U. maydis* infected maize tissue treated with HA-specific antiserum (A1). *U. maydis* hyphae of strain SG200 (stained by WGA-AF633) were not detected (overlay, A2). B: Confocal projection showing immunodetection of plant tubulin in *U. maydis* infected maize tissue treated with a tubulin specific antibody (B1). *U. maydis* hyphae of strain SG200 (stained by WGA-AF633) were not detected by tubulin specific serum (overlay, B2).
(6.44 MB TIF)

Table S1 Maize genes with significant changes in expression in response to infection with *U. maydis* strain SG200Δpep1 24 hpi compared to mock infected plants.
(0.08 MB XLS)

Table S2 Maize genes with significant changes in expression in response to infection with *U. maydis* strain SG200Δpep1 compared to infection with strain SG200 24 hpi.
(0.05 MB XLS)

Table S3 Disease rating of Early Golden Bantam maize plants 12 dpi with *U. maydis* strains used in this study.
(0.02 MB XLS)
