# Supplementary figures and images for: Pep1, a Secreted Effector Protein of Ustilago maydis, Is Required for Successful Invasion of Plant Cells
Source: PLoS Pathog. 2009 Feb 6;5(2):e1000290. doi: 10.1371/journal.ppat.1000290 (PMC2631132; doi:10.1371/journal.ppat.1000290)

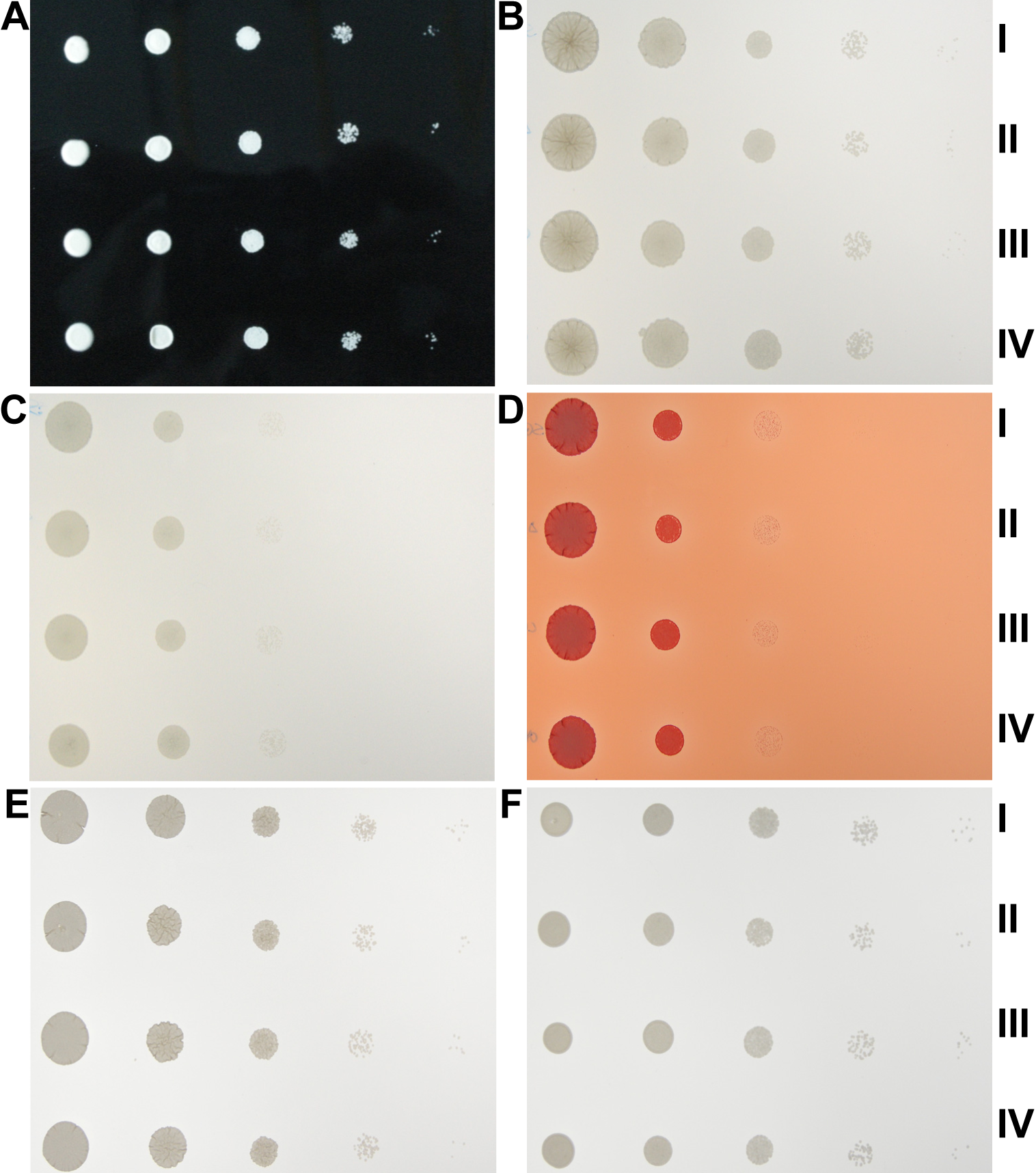

Supplement: Figure S1 — Growth of U. maydis SG200, SG200Δpep1 and SG200Δpep1otef:pep1 on growth media providing different stresses. Precultures of U. maydis were grown in YEPSL to an OD600 of 1.0. Cells were washed in water and recalibrated to an OD600 of 1.0 and diluted 10-fold each in four steps. From these suspensions droplets of 6 µl each were dropped on the different media. After 48 hours incubation at 28°C pictures were taken. A: PD agar containing 1% Charcoal; B: CM agar supplied with Calcofluor (100 µg/ml); C: CM agar supplied with 2 mM H2O2; D: CM agar supplied with Congored (50 µg/ml); E: Ammonium Minimal Medium; F: Nitrogen Minimal medium. I) SG200 II) SG200Δpep1 III) SG200Δpep1-pep1 IV) SG200Δpep1-otef:pep1. (7.83 MB TIF) [file ppat.1000290.s001.tif]

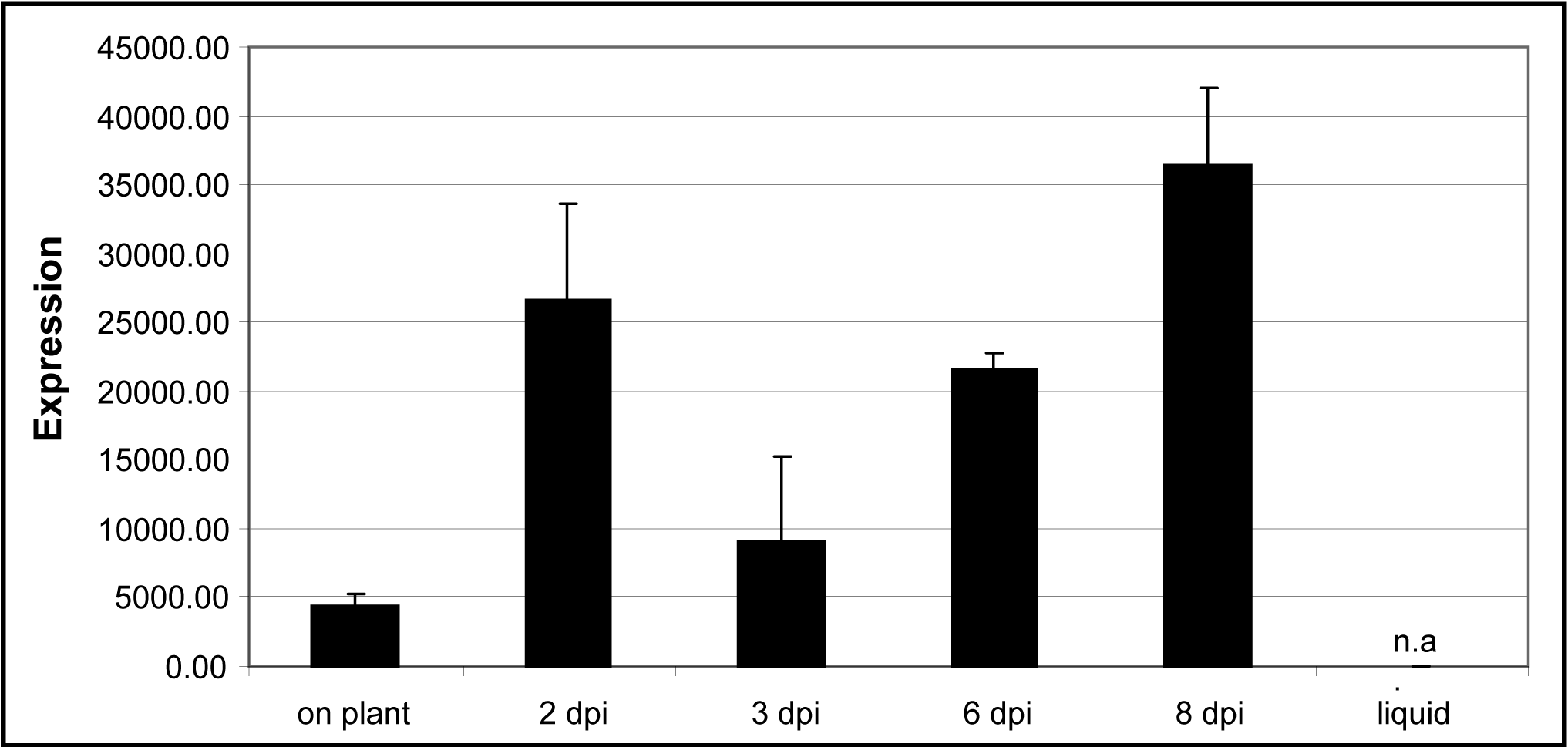

Supplement: Figure S2 — Expression of pep1 during pathogenic development of U. maydis. Quantitative real-time PCR on pep1 expression of U. maydis strain SG200. Sporidia grown in axenic culture did not show detectible expression of pep1. In SG200 cells that were extracted from the maize leaf surface (18 hpi) pep1 transcript was detected. High levels of pep1 expression were detected in maize leaf tissue taken at different time points after infection with SG200. The strongest expression of pep1 was observed during the early biotrophic phase (2 dpi) and during late stages of infection (6 and 8 dpi). (5.94 MB TIF) [file ppat.1000290.s002.tif]

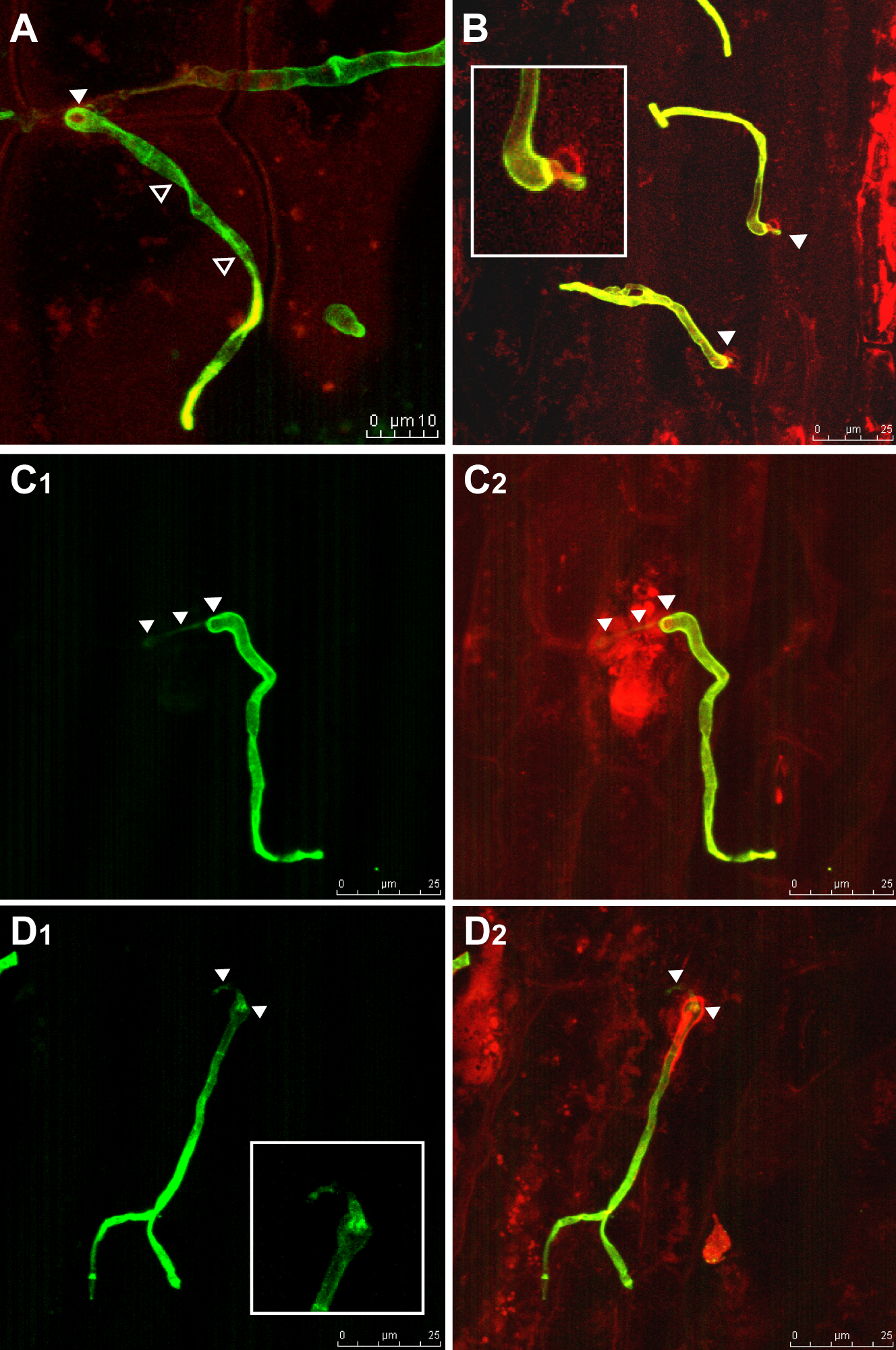

Supplement: Figure S3 — Microscopic analysis of U. maydis FB1/2Δpep1 mutants after inoculation on maize plants. Confocal projections showing fungal hyphae stained with WGA-AF488 (green) and plant cells stained with propidium-iodide (red) 24 hpi. A: FB1×FB2 crossings have penetrated the leaf surface (white arrowhead) and grow intracellularly. Hyphae on the leaf surface are collapsed (open arrowheads) after plant penetration. B–D: At the same time-point, the FB1Δpep1×FB2Δpep1 dikaryon was arrested immediately upon penetration similar to SG200Δpep1 (Figure 2). In addition, short hyphae of FB1Δpep1×FB2Δpep1 (left panel, C1 and D1 and insert) can be found in collapsed epidermis cells (overlay: right panel, C21 and D2). Bars are given. (7.99 MB TIF) [file ppat.1000290.s003.tif]

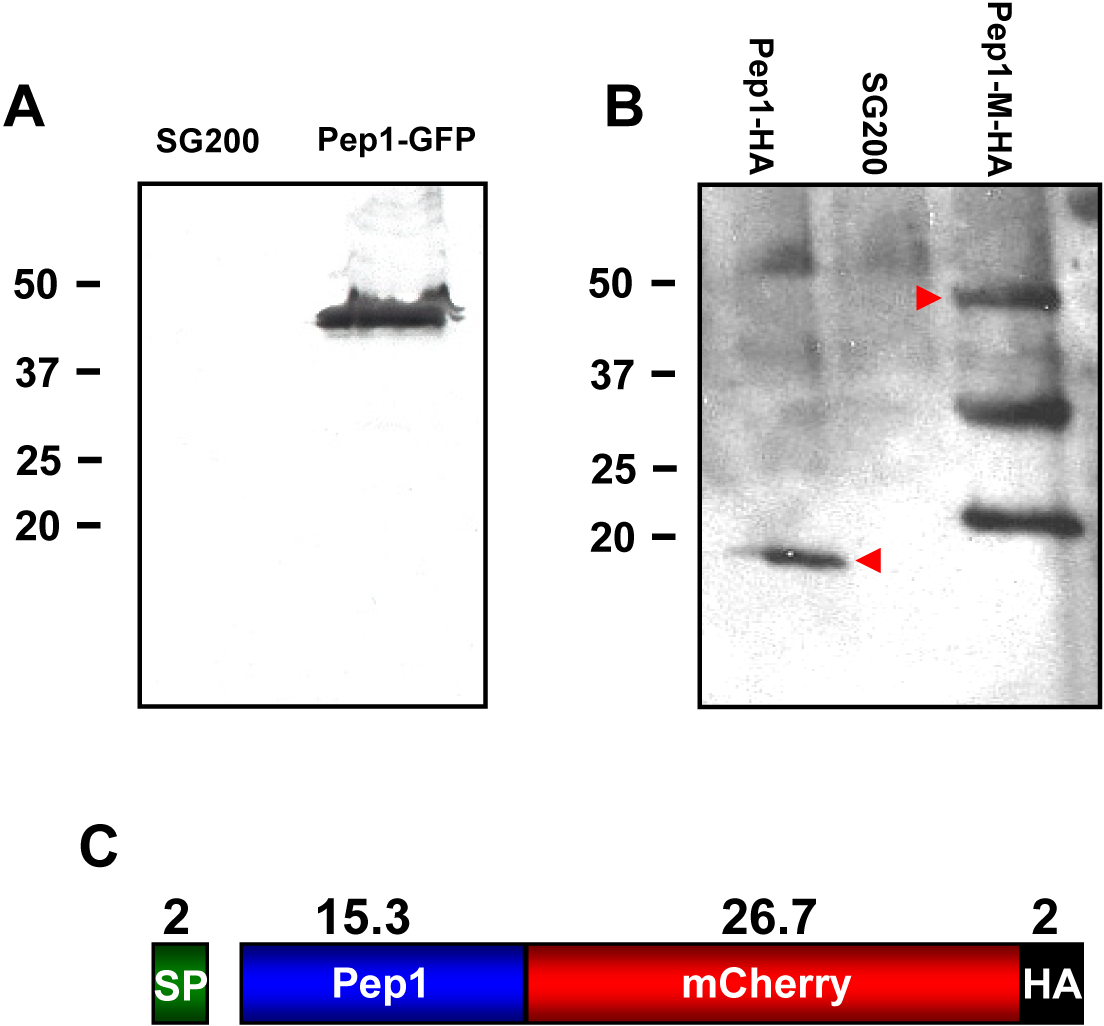

Supplement: Figure S4 — Western detection of Pep1-GFP, Pep1-HA and Pep1-mCherry-HA. Western blot of Pep1-GFP secreted from U. maydis strain SG200Δpep1oma:pep1-gfp. SG200: In culture-supernatant of SG200 cells, no Pep1-GFP was detected by an anti-GFP serum. Pep1-GFP: In culture-supernatant of SG200Δpep1oma:pep1-gfp, Pep1-GFP was detected in full-length. B: Immunoprecipitation of Pep1-HA and Pep1-mCherry-HA (Pep1-MHA): HA tagged Pep1 and Pep1-mCherry were immunoprecipitated from maize tissue infected with U. maydis strain SG200Δpep1-pep1HA and SG200Δpep1-pep1MHA, respectively (3 dpi) using monoclonal HA-specific antibodies. SG200: From SG200 infected maize tissue, no precipitated protein was detected. Red arrows: Full length fusion protein at the expected size for Pep1-HA and Pep1-mCherry-HA. C: Schematical description of the Pep1-mCherry-HA fusion protein. Numbers: Expected molecular weight [kDa] of the individual parts of the fusion protein. SP: signal peptide (cleaved off during secretion). (3.43 MB TIF) [file ppat.1000290.s004.tif]

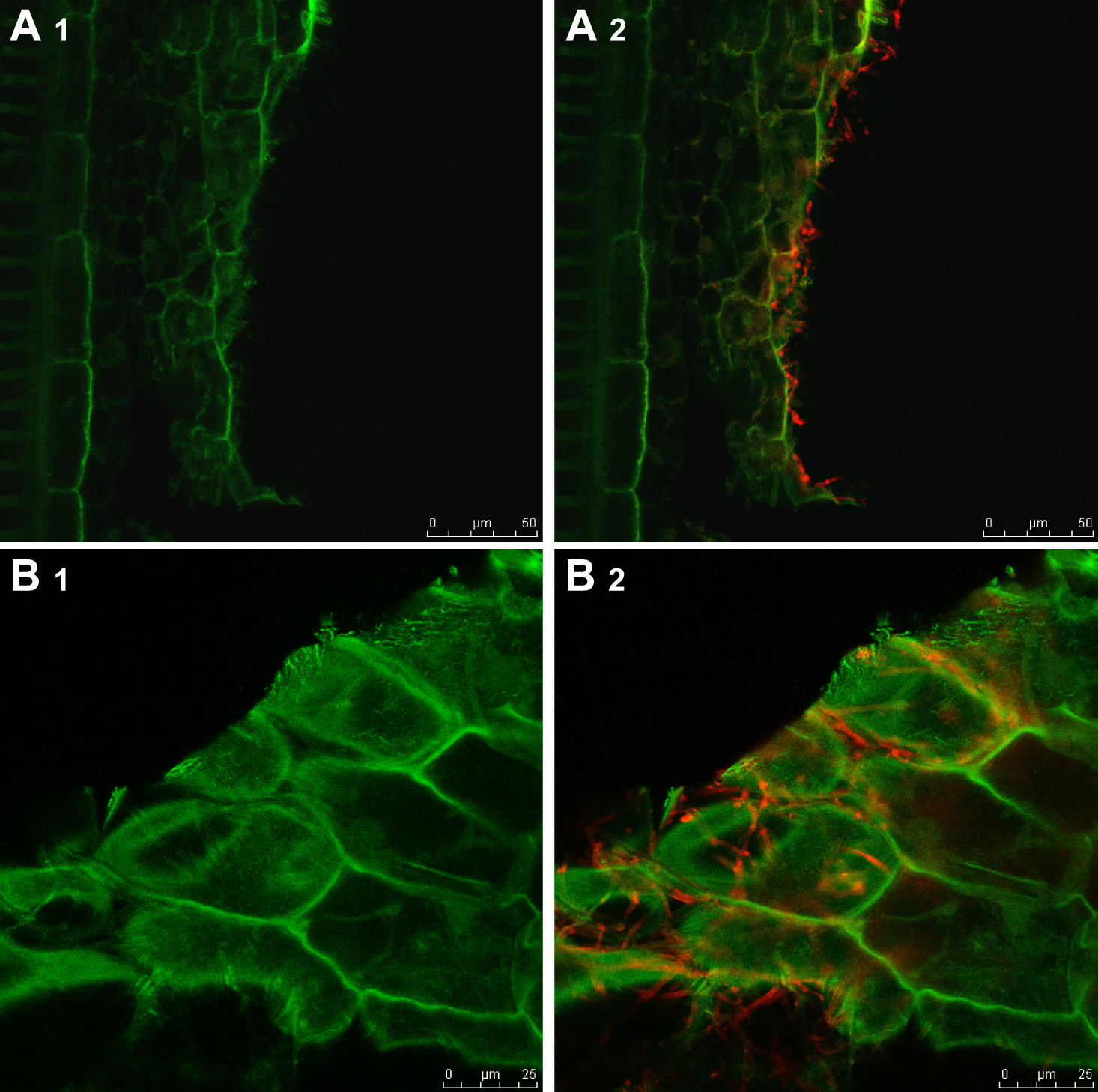

Supplement: Figure S5 — Control samples showing specificity of anti-HA serum used for immunolocalization of Pep1-HA. A: Confocal projection showing unspecific fluorescence of U. maydis infected maize tissue treated with HA-specific antiserum (A1). U. maydis hyphae of strain SG200 (stained by WGA-AF633) were not detected (overlay, A2). B: Confocal projection showing immunodetection of plant tubulin in U. maydis infected maize tissue treated with a tubulin specific antibody (B1). U. maydis hyphae of strain SG200 (stained by WGA-AF633) were not detected by tubulin specific serum (overlay, B2). (6.44 MB TIF) [file ppat.1000290.s005.tif]
